# Shikonin induces ROS-based mitochondria-mediated apoptosis in colon cancer

**DOI:** 10.18632/oncotarget.22618

**Published:** 2017-11-17

**Authors:** Wenquan Liang, Jianxin Cui, Kecheng Zhang, Hongqing Xi, Aizhen Cai, Jiyang Li, Yunhe Gao, Chong Hu, Yi Liu, Yixun Lu, Ning Wang, Xiaosong Wu, Bo Wei, Lin Chen

**Affiliations:** ^1^ Department of General Surgery, Chinese People’s Liberation Army General Hospital, Beijing 100853, China; ^2^ Institute of General Surgery, Chinese People’s Liberation Army General Hospital, Beijing 100853, China

**Keywords:** colon cancer, shikonin, ROS, mitochondria, apoptosis

## Abstract

Colon cancer is the third most common malignancy worldwide, and chemotherapy is a widely used strategy in clinical therapy. Chemotherapy-resistant of colon cancer is the main cause of recurrence and progression. Novel drugs with efficacy and safety in treating colon cancer are urgently needed. Shikonin, a naphthoquinone derived from the roots of the herbal plant *Lithospermum erythrorhizon*, has been determined to be a potent anti-tumor agent. The aim of the present study was to detect the underlying anti-tumor mechanism of shikonin in colon cancer. We found that shikonin suppressed the growth of colon cancer cells in a dose-dependent manner *in vitro* and *in vivo*. Shikonin induced mitochondria-mediated apoptosis, which was regulated by Bcl-2 family proteins. Shikonin increased the generation of intracellular ROS, which played an upstream role in shikonin-induced apoptosis. Our data indicated that generation of ROS, down-regulated expression of Bcl-2 and Bcl-xL, depolarization of the mitochondrial membrane potential and activation of the caspase cascade were components of the programmed event of shikonin-induced apoptosis in colon cancer cells. In addition, shikonin presented minimal toxicity to non-neoplastic colon cells and no liver injury in xenograft models, showing safety in the control of colon cancer cell growth *in vitro* and *in vivo*. Taken together, our findings suggest that shikonin might serve as a potential novel therapeutic drug in the treatment of human colon cancer.

## INTRODUCTION

Colon cancer is considered one of the most commonly diagnosed cancers worldwide. The incidence of colon cancer is high in developed countries, reflecting a prevalence of risk factors, including an unhealthy diet and obesity [1]. However, in the last few years, increasing attention has been focused on many developing areas with growing populations and increasingly westernized lifestyles [2]. Although surgical resection is mainly a curative therapy in the early stage, many patients are in an advanced stage at the time of diagnosis due to the lack of colon cancer screening [3, 4]. Chemotherapy is a commonly used strategy in colon cancer. However, many patients eventually relapse and develop drug resistance [5, 6]. On this account, searching for novel drugs with efficacy and safety in the treatment of colon cancer is of utmost importance.

Shikonin is a naphthoquinone derived from the roots of the herbal plant *Lithospermum erythrorhizon*, which has anti-viral, anti-carcinogenic, anti-microbial and anti-inflammatory properties [7]. Previous studies have reported that shikonin exerts anti-tumor effects in multiple tumors, such as inhibiting melanoma proliferation [8], inducing apoptosis in acute myeloid leukemia [9] and activating necroptosis in breast cancer [10]. Our previous report also demonstrated that shikonin induced mitochondria-mediated apoptosis and enhanced the chemotherapeutic sensitivity of gastric cancer [11]. In studies of colon cancer, shikonin was reported to enhance cisplatin-induced cancer cells apoptosis [12]. The efficacy and safety of shikonin in treating colon cancer require further studies.

Cell apoptosis, also called programmed cell death, plays an important role in the control of development, cell proliferation and stress responses [13]. Tumorigenesis is associated with changes in apoptotic gene expression and involves apoptotic pathways [14]. The underlying mechanism of tumor drug-resistance has largely been attributed to anti-apoptotic pathways and the sensitivity of cancer cells to chemotherapeutic drug-induced apoptosis depends on the balance between pro-apoptotic and anti-apoptotic signals. The death receptor-mediated pathway and mitochondria-mediated pathway are two major identified apoptotic pathways [15]. Shikonin has been identified as an apoptosis inducer in many tumors. Additionally, we previously reported that shikonin induced caspase-dependent and caspase–independent apoptosis in gastric cancer cells [11]. Further studies of shikonin-induced apoptosis in colon cancer are needed for better understanding.

Reactive oxygen species (ROS) and tumor biology are intertwined [16]. On the one hand, oxidative stress is required for tumor growth, metastasis and dissemination; on the other hand, increased basal oxidative stress makes tumors more vulnerable to chemotherapeutic agents. The accumulation of intracellular ROS causes damage to proteins, enzymes and membranes of organelles, which finally activates apoptosis signaling pathways [17]. Therefore, increasing oxidative stress is a novel therapeutic strategy to selectively kill cancer cells.

In the present study, our data showed that shikonin effectively suppressed the proliferation of colon cancer cells and blocked the cell cycle in G1 phase. Shikonin activated the caspase cascade and induced apoptosis in colon cancer cells *in vitro*. Mitochondrial dysfunction is involved in shikonin-induced apoptosis, which is controlled by Bcl-2 family proteins. Shikonin is a ROS inducer in colon cancer cells and the accumulation of intracellular ROS plays an upstream role in mitochondria-mediated apoptosis. In addition, shikonin exerts good safety and efficacy in inducing antitumor responses of colon cancer *in vivo*. In conclusion, our studies elucidated the mechanism of shikonin-induced apoptosis in colon cancer *in vitro* and *in vivo*, and demonstrated that shikonin might be a candidate drug for colon cancer therapy.

## RESULTS

### Shikonin suppresses the proliferation of colon cancer cells *in vitro*

**Figure 1:**
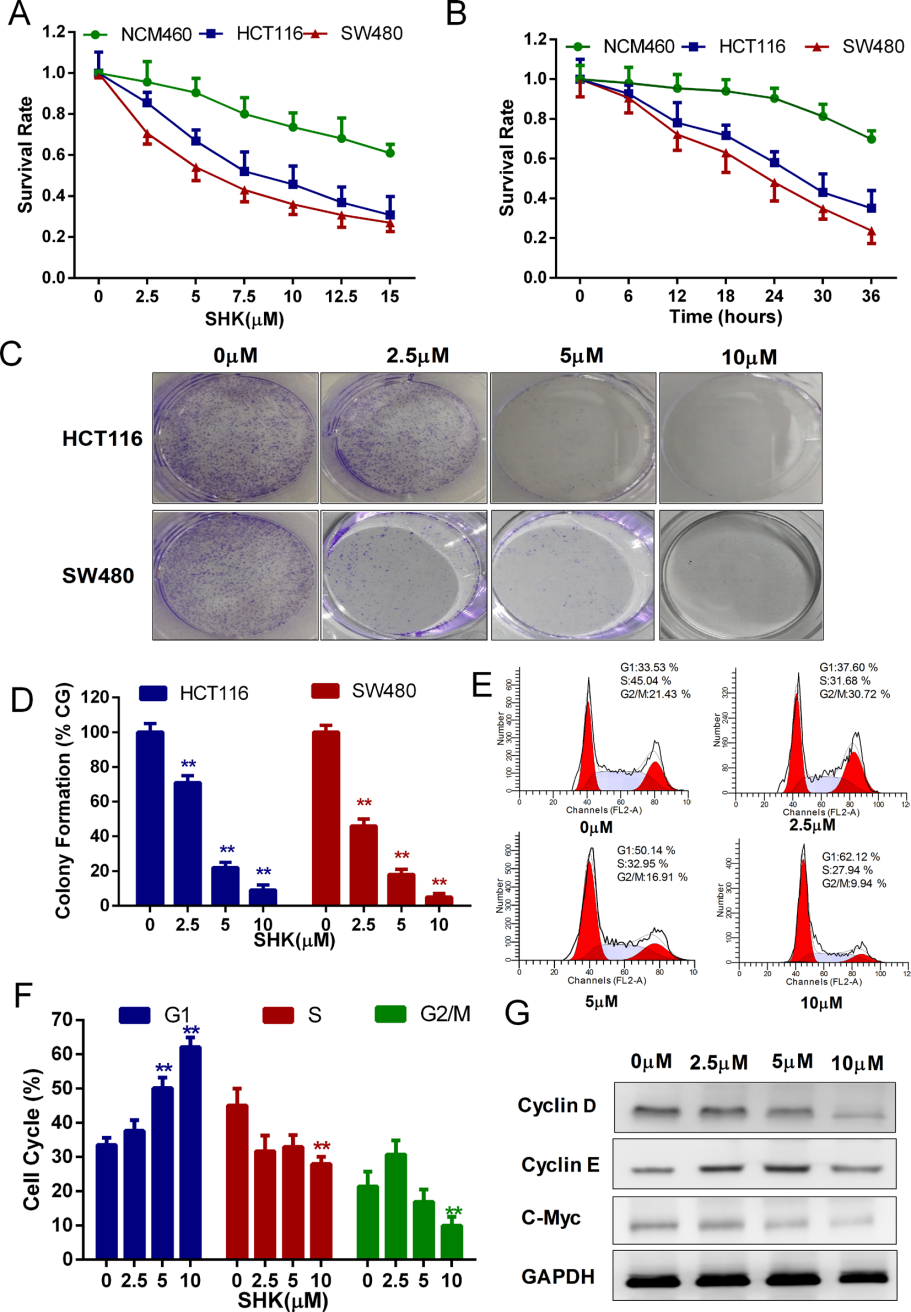
Shikonin suppresses the proliferation of colon cancer cells *in vitro* **(A)** Cells were treated with increasing concentrations of shikonin for 24 h. **(B)** Cells were treated with 5 μM shikonin for different times as indicated. The cell viability of (A) and (B) was determined by the MTT assay. **(C)** Clonogenic assays of colon cancer cells were pre-treated with increasing concentrations of shikonin for 24 h before replacing the culture medium, followed by culturing for 7 days. Quantification of clone formation is shown in **(D)**. **(E)** Cell cycle analysis of SW480 cells was performed by FACS in the presence of propidium iodide buffer. Quantification of the distribution of the three distinct phases of the cell cycle (G1, S and G2/M phase) is shown in **(F)**. **(G)** Western blot analysis of Cyclin D, Cyclin E and c-Myc detected the dose-dependent effect of shikonin acting on SW480 cells for 24 h. Assays were performed in triplicate. ^*^ < 0.05, ^**^ < 0.01.

Previous studies have confirmed that shikonin inhibited cell proliferation in different cancer cells with different concentrations [12, 18, 19]. To identify the optimum drug level of shikonin in colon cancer cells, we treated the human colon cancer cell lines HCT116, SW480 and the human normal colon mucosal epithelial cell line NCM460 with a concentration gradient of shikonin (Figure 1A). Shikonin suppressed the proliferation of colon cancer cells in a dose-dependent manner. However, shikonin displayed higher suppressive activities on human colon cancer cell lines HCT116 and SW480 than the human normal colon mucosal epithelial cell line NCM460, indicating the selectivity of shikonin in killing tumor cells. We incubated cells in the medium containing shikonin for different time courses to determine the time-dependent effects (Figure 1B). The toxicity of shikonin increased time-dependently, and the human normal colon mucosal epithelial cell line NCM460 was less sensitive to the increasing incubation time than the human colon cancer cell lines HCT116 and SW480. In addition, shikonin had a higher inhibition rate in SW480 cancer cells than in HCT116 cancer cells. In the following study, we performed clonogenic survival assays to examine the ability of shikonin in clone formation (Figure 1C-1D). Cloning efficiency was reduced in the presence of shikonin in HCT116 and SW480 cancer cells. Flow cytometric analysis of DNA showed a significant increase in the percentage of the G1 phase peak in SW480 cells processed by a concentration gradient of shikonin for 24 h (Figure 1E-1F). Cyclin D and Cyclin E are associated with G1 cycle progression. We found Cyclin D expression was decreased with shikonin treatment, whereas Cyclin E expression did not show specific change (Figure 1F). The level of c-Myc, which regulated Cyclins, also decreased with shikonin treatment (Figure 1F). These data suggested that shikonin suppressed the proliferation of colon cancer cells in a dose- and time-dependent manner and induced cell growth inhibition by the induction G1 cell cycle arrest.

### Shikonin induces apoptosis in colon cancer cells *in vitro*

**Figure 2:**
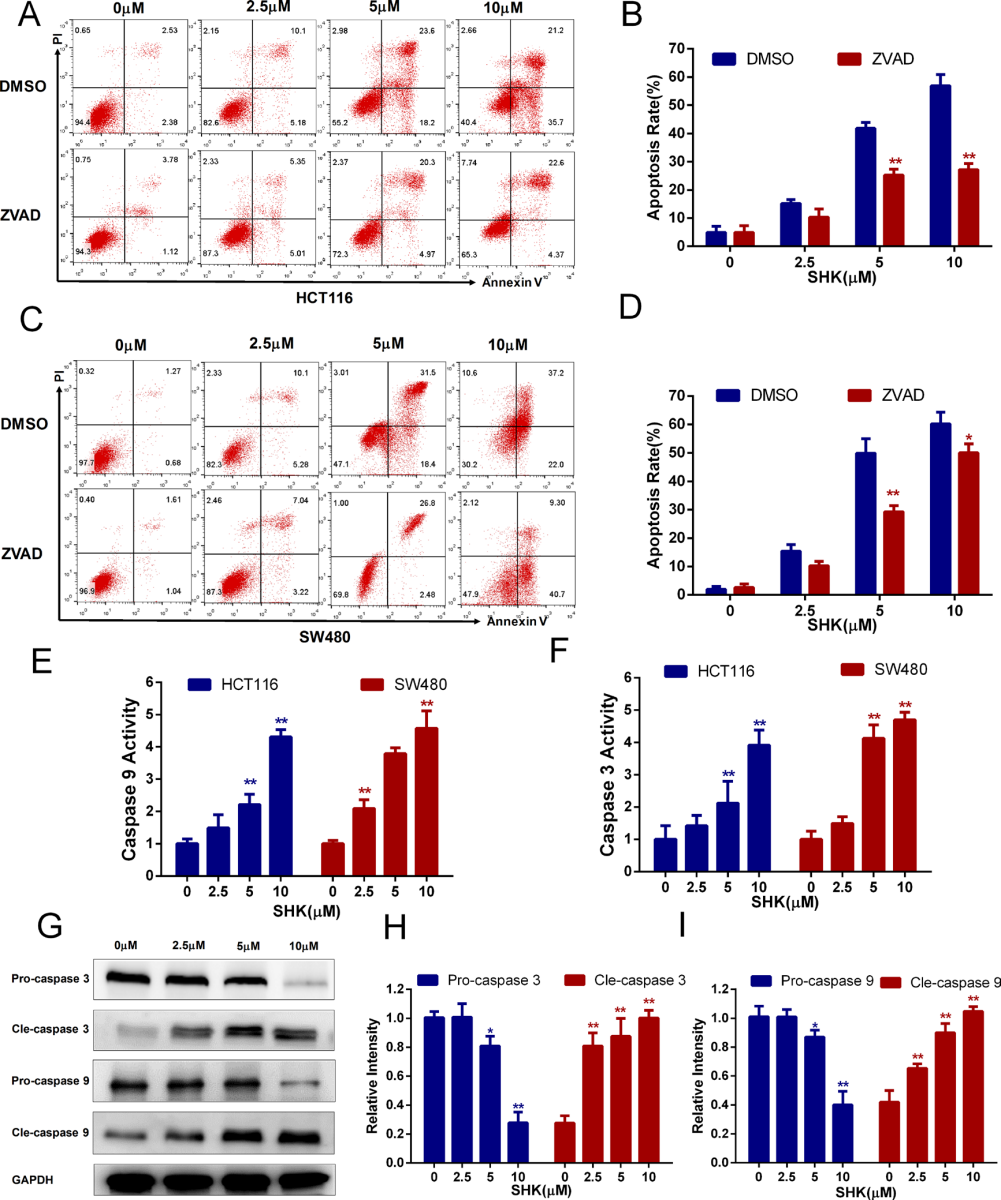
Shikonin induces apoptosis in colon cancer cells *in vitro* **(A-B)** Shikonin induced apoptosis in HCT116 cells. After the treatment of shikonin at different concentrations with or without the co-treatment of ZVAD (20 μM) for 24 h, cell death was observed by flow cytometry using PI and Annexin V staining. Quantification of the apoptosis rate of HCT116 cells is shown in (B). **(C-D)** Shikonin induced apoptosis in SW480 cells. Treatment was the same as that in (A). Quantification of the apoptosis rate of SW480 cells is shown in (D). **(E-F)** Colon cancer cells of HCT116 and SW480 were treated with shikonin for 24 h, and then caspase 9 and 3 activities of cell lysis were determined using assay kits. **(G-I)** Western blot analysis of caspase 3 and 9 detected the dose-dependent effect of shikonin acting on SW480 cells for 24 h. Assays were performed in triplicate. ^*^ < 0.05, ^**^ < 0.01.

It has been confirmed that shikonin induced both apoptotic and necroptotic cell death in some cancer cells [20, 21]. To identify the cell death type induced by shikonin in colon cancer cells, we quantitatively examined the death-promoting effects of shikonin using a two-color analysis with an Annexin V-FITC and PI staining kit. After treatment with a concentration gradient of shikonin, HCT116 (Figure 1A-1B) and SW480 (Figure 2C-2D) cells were induced a marked increase in the proportion of early (Annexin V+, PI-) and late apoptotic (Annexin V+, PI+) cells, and there were almost no necroptotic (Annexin V-, PI+) cells in HCT116 and SW480 colon cancer cells. ZVAD is a type of pan-caspase inhibitor capable of increasing the necroptotic proportion if shikonin could induce necroptosis in colon cancer cells. However, we did not identify the necroptotic proportion increasing phenomenon in HCT116 and SW480 cells. Moreover, the proportion of early apoptotic cells (Annexin V+, PI-) decreased with co-treatment of ZVAD, indicating that shikonin had the ability to induce apoptosis. Caspase 9 and 3 are highly inducible caspases that controll cell apoptosis [22, 23]. We measured the caspase 9 and 3 activities of cell lysate and found that the activities of caspase 9 and 3 increased with increasing amounts of shikonin (Figure 2E-2F). The cleavage of caspases is a marker of activation. Western blot analysis of caspase 3 and 9 detected dose-dependent effects on caspase cleavage in shikonin-induced apoptosis (Figure 2G-2I). Taken together, these data suggested that shikonin activated the caspase cascade and induced apoptosis in colon cancer cells *in vitro*.

### Shikonin-induced apoptosis in colon cancer cells is mediated by mitochondria

**Figure 3:**
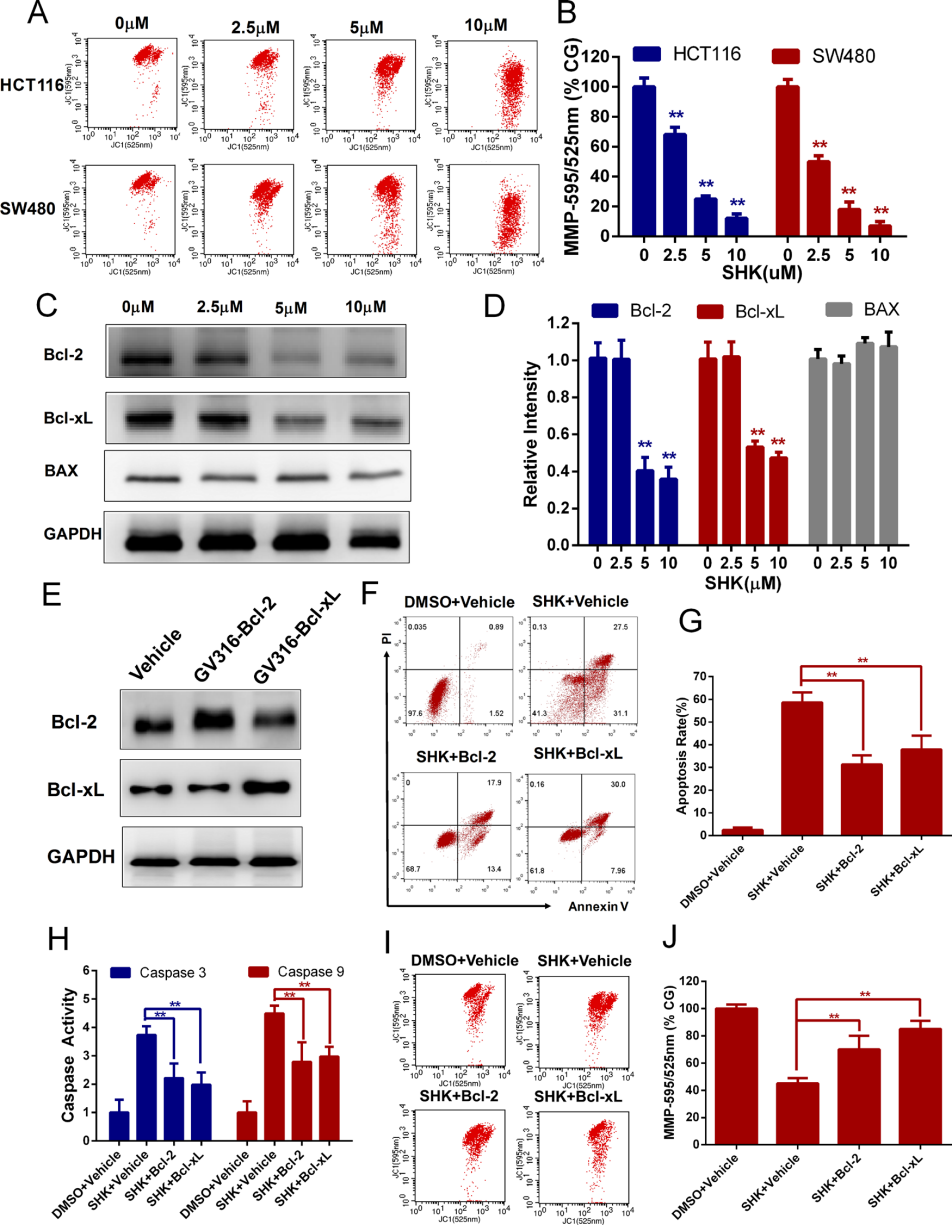
Shikonin-induced apoptosis in colon cancer cells is mitochondrial mediated **(A-B)** Mitochondrial membrane potential (Δψ) analysis of HCT116 and SW480 cells to detect the dose-dependent effect of shikonin acting on mitochondrial dysfunction. The mitochondrial membrane potential was detected by flow cytometry using JC-1 probes 12 h after SHK treatment. CG indicates the control group. **(C-D)** Bcl-2, Bcl-xL and BAX expression in SW480 cells treated with shikonin for 24 h was analyzed by Western blot. GAPDH served as the loading control. **(E)** Western blot analysis of Bcl-2 and Bcl-xL to detect the overexpression effect of SW480 cells transiently transfected with overexpressing vectors (GV316). **(F-G)** Effects of Bcl-2 or Bcl-xL overexpression on shikonin-induced apoptosis in SW480 cells. Cells were treated with 5 μM shikonin for 24 h, and cell death was detected by flow cytometry using PI and annexin V staining. **(H)** Caspase 9 and 3 activity changes following Bcl-2 or Bcl-xL overexpression in SW480 cells. Cells were treated with 5 μM shikonin for 24 h, and caspase activities were determined by assay kits. **(I-J)** Mitochondrial membrane potential analysis of SW480 cells treated with 5 μM shikonin for 12 h after transient transfection with Bcl-2 or Bcl-xL. Assays were performed in triplicate. ^*^ < 0.05, ^**^ < 0.01.

Apoptosis is the process of programmed cell death and the mitochondrial apoptosis-induced channel is an early marker of the onset of apoptosis [24, 25]. There are differences in voltage across the mitochondrial membrane, known as the mitochondrial membrane potential (MMP or Δψ)[26]. The mitochondrial membrane potential was detected via flow cytometry using JC-1 probes. JC-1 aggregates and forms JC-polymers, that emit red fluorescence at 595 nm, in polarized mitochondria. In contract, JC-1 exists as monomers, which emit green fluorescence at 525 nm, in depolarized mitochondria. The mitochondrial membrane potential could be indicated by the 595/525-nm fluorenscence intensity ratio. With the treatment of a concentration gradient of shikonin, the mitochondrial membrane potentials of HCT116 and SW480 cells were depolarized (Figure 3A-3B). Members of the Bcl-2 family regulate apoptosis by regulating the formation of mitochondrial apoptosis-induced channels [27]. Bcl-2 and Bcl-xL were restraining apoptosis proteins and BAX was promoting apoptosis protein. We detected the expressions of Bcl-2 and Bcl-xL and found a downward trend with the treatment of shikonin, however we did not found significant expression change in BAX (Figure 3C-3D). To further elucidate the mechanisms of the Bcl-2 family in regulating shikonin-induced apoptosis, Bcl-2 or Bcl-xL was transiently transfected using overexpression vectors and the overexpression was confirmed by western blot (Figure 3E). The cell apoptosis rates were reduced in Bcl-2 or Bcl-xL overexpressing cells treated with shikonin (Figure 3F-3G). Moreover, the shikonin-induced increase in caspase 9 and 3 activities diminished (Figure 3H) and shikonin-induced depolarization of the mitochondrial membrane potential was attenuated (Figure 3I-3J) when Bcl-2 or Bcl-xL was overexpressed. These data suggested that shikonin induced apoptosis via the activation of the mitochondrial pathway in colon cancer cells.

### Shikonin-induced apoptosis in colon cancer cells depends on the accumulation of intracellular ROS

**Figure 4:**
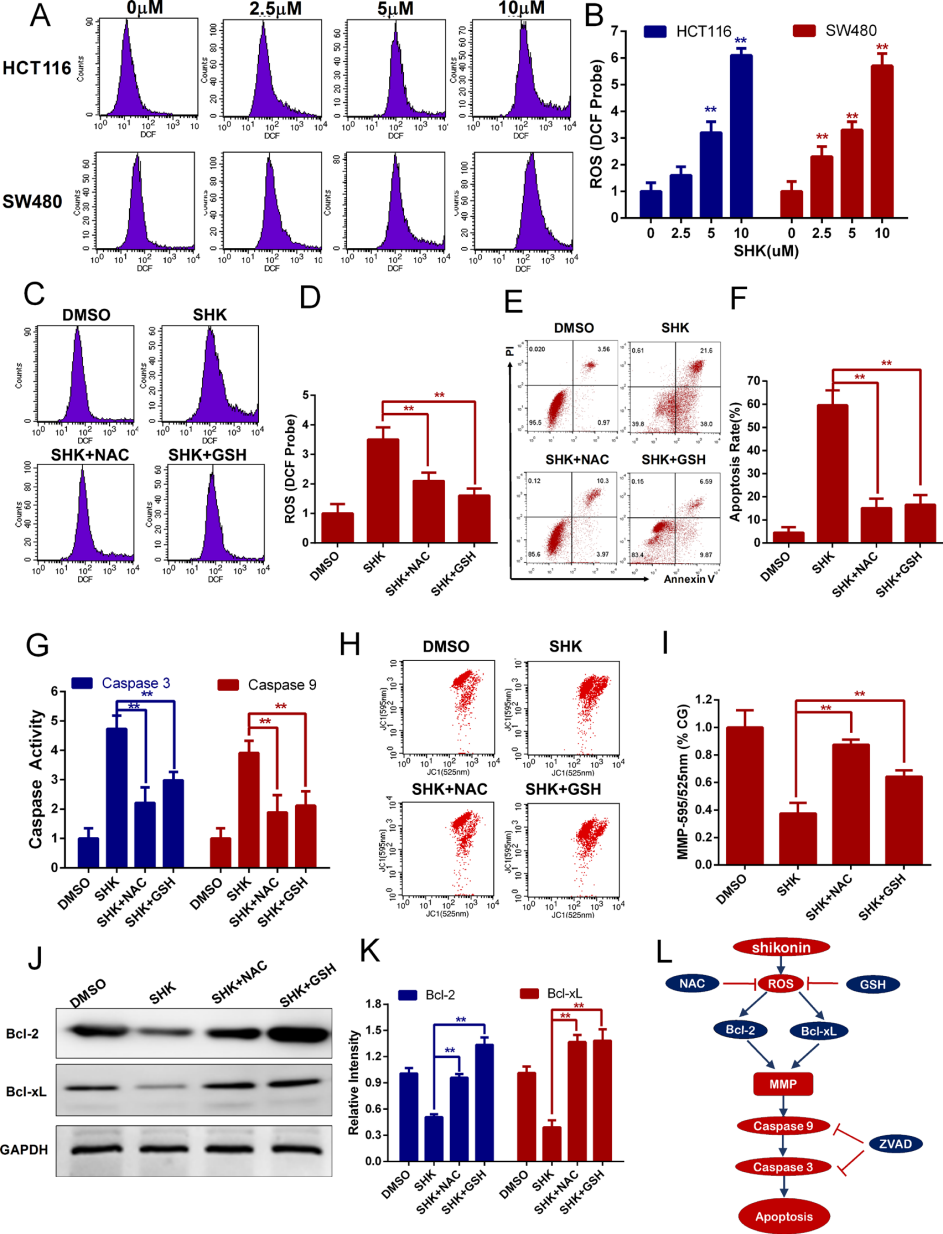
Shikonin induces apoptosis in colon cancer cells by the accumulation of intracellular ROS **(A-B)** Intracellular ROS of HCT116 and SW480 cells was monitored by DCFH-DA probes to detect the dose-depended effect of shikonin treatment for 12 h. ROS was detected by flow cytometry. **(C-D)** ROS scavengers NAC (1 mM) and GSH (1 mM) were added to examine the influence of ROS accumulation induced by shikonin (5 μM) treatment for 12 h in SW480 cells. **(E-F)** Shikonin-induced apoptosis in SW480 cells was attenuated by ROS scavengers NAC (1 mM) and GSH (1 mM). Cells were treated with 5 μM shikonin for 24 h, and cell death was detected by flow cytometry using PI and Annexin V staining. **(G)** Caspase 3 and 9 activities were diminished by ROS scavengers NAC (1 mM) and GSH (1 mM) in SW480 cells treated with 5 μM shikonin for 24 h. **(H-I)** Mitochondrial membrane potential analysis of SW480 cells co-treated with 5 μM shikonin and ROS scavengers NAC (1 mM) or GSH (1 mM) for 12 h. The mitochondrial membrane potential was detected by flow cytometry using JC-1 probes, and CG indicates the control group. **(J-K)** Bcl-2 and Bcl-xL expression of SW480 cells treated with 5 μM shikonin and ROS scavengers NAC (1 mM) or GSH (1 mM) for 24 h was analyzed by Western blot. GAPDH served as loading control. **(L)** Pathway of SHK-induced apoptotic cell death in colon cancer cells.

The accumulation of intracellular ROS could be generated not only by abnormal metabolism but also by exogenous sources of drugs. ROS contain reactive chemical oxygen that could activate mitochondria-dependent apoptosis [28–30]. Previous studies have reported that shikonin induces the accumulation of ROS in some tumor cells [12, 31]. Intracellular ROS could oxidize non-fluorescing DCFH probes to fluorescent DCF probes that could be detected by flow cytometer. Therefore, we evaluated the production of ROS in HCT116 and SW480 cells with shikonin treatment and found that shikonin dose-dependently increased the generation of intracellular ROS in colon cancer cells (Figure 4A-4B). N-acetyl-L-cysteine (NAC) and L-glutathione (GSH) are two conventional antioxidants. We measured the level of intracellular ROS with co-treatment of shikonin and antioxidants in colon cancer cells. NAC or GSH could block the shikonin-dependent burst of intracellular ROS (Figure 4C-4D). In the following studies, we detected the effects of the antioxidants NAC or GSH in shikonin-induced apoptosis and mitochondrial dysfunction. The cell apoptosis rates were noticeably decreased with co-treatment of the antioxidants NAC or GSH (Figure 4E-4F). Caspase 3 and 9 activities were attenuated dramatically in colon cancer cells co-treated with antioxidants NAC or GSH (Figure 3G). Meanwhile, shikonin-induced depolarization of mitochondrial membrane potential was attenuated (Figure 4H-4I) and decreased Bcl-2 or Bcl-xL expression also recovered (Figure 4J-4K) with co-treatment with the antioxidants NAC or GSH in colon cancer cells. Taken together, shikonin induces apoptosis in colon cancer cells by generating intracellular ROS and activating the mitochondrial pathway as shown in Figure 4L.

### Shikonin exerts anticancer activities and induces apoptosis of xenografts *in vivo*

**Figure 5:**
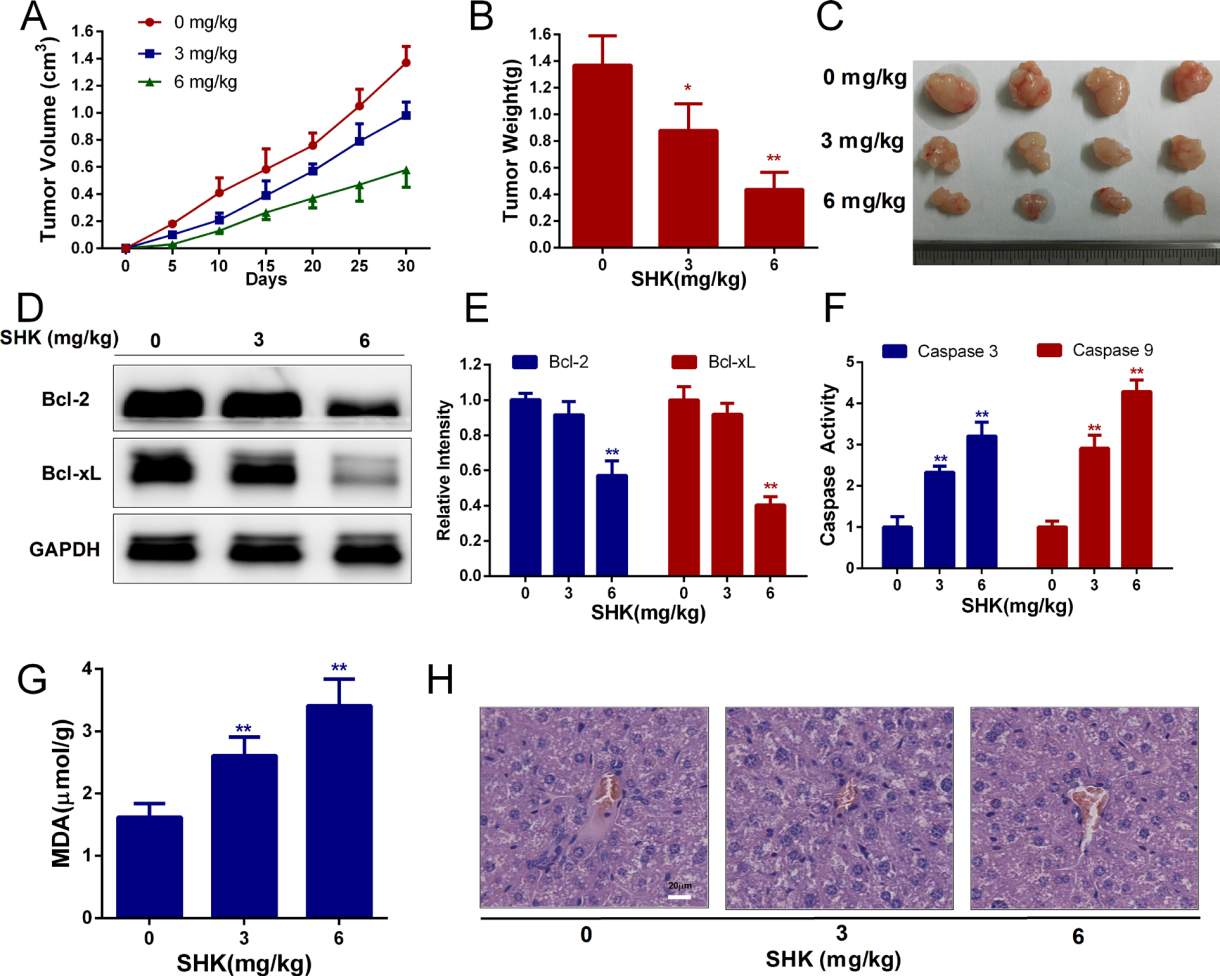
Shikonin exerts anticancer activities and induces the apoptosis of xenografts *in vivo* **(A)** The tumor volume of xenografts was measured every 5 days after the transplantation of SW480 cancer cells in nude mice. **(B)** Tumor weight of xenografts after treatment with shikonin (0, 3, 6 mg/kg body weight) for 30 days. **(C)** Tumor pictures of xenografts treated with shikonin. **(D-E)** Bcl-2 and Bcl-xL expression of xenografts after treatment with shikonin for 30 days was analyzed by Western blot. GAPDH served as the loading control. **(F)** Caspase 3 and 9 activities of xenografts were determined by assay kits. **(G)** The MDA content indicated that lipid peroxidation was detected. **(H)** Histological toxicity analysis of the liver (200×, Scale bars: 20 μm) in nude mice by hematoxylin and eosin staining after shikonin treatment (0, 3, 6 mg/kg body weight).

To further determine the anticancer activities of shikonin, we established xenograft models bearing SW480 colon cancer cells in immuno-deficient mice. Cells were subcutaneously implanted and a concentration gradient of shikonin was administered via gavage. The tumor volumes of the xenografts were measured and colon tumor growth was effectively inhibited in nude mice (Figure 5A). After 30 days of administration, the mice were sacrificed and treatment with shikonin was found to have significantly inhibited increases in tumor weight (Figure 5B-5C). Moreover, shikonin also decreased Bcl-2 and Bcl-xL expression (Figure 5D-5E) and enhanced caspase 3 and 9 activities (Figure 5F) of xenografts *in vivo*, suggesting that shikonin induces mitochondria-mediated apoptosis in animal models. Malondialdehyde (MDA) is one of the breakdown products of lipid peroxidation and a marker of oxidative damage. The MDA content was measured and showed a tendency to rise with shikonin treatment (Figure 5G), suggesting that shikonin increases oxidative stress in animal models. In addition, the liver histological toxicity of nude mice was identified and no significant liver injury was found with shikonin treatment *in vivo* (Figure 5H). These results suggested that shikonin was efficient at inducing the antitumor responses of colon cancer *in vivo*.

## DISCUSSION

Cancer is a major public health problem worldwide. Colon cancer is the third most common malignancy both in terms of estimated new cases and estimated deaths in 2016 [32]. Although enormous efforts have been made in early diagnosis and therapy, many patients have died of drug-resistant recurrence and progress in improving overall survival has been relatively slow [33]. The development of novel agents offers an alternative strategy against drug-resistance in colon cancer. Shikonin has been reported to have anti-tumor and sensitizing effects in some solid tumors [11, 34, 35]. In present study, we found that shikonin suppressed the proliferation of colon cancer cells in a dose-dependent manner both *in vitro* and *in vivo*. Cell cycle inhibition is a common mechanism proposed for the anticancer effects of shikonin [10]. We analyzed the cell cycle distribution of colon cancer cells which showed a significant G1 phase arrest with treatment using a gradient concentration of shikonin. Previous studies have determined that shikonin showed minimal toxicity for non-neoplastic cells, such as the immortalized gastric epithelial cell line GES-1 [11] and normal human hepatic cell line L02 [36]. In our studies, we also found that immortalized but nontumorigenic colon NCM 460 cells showed strong resistance to shikonin compared with colon cancer cells. In addition, we found no evidence of significant liver injury from shikonin treatment in mice. The evidence showed that shikonin was efficacious and safe for the growth control of colon cancer cells, demonstrated the potential utility of shikonin in clinical settings.

Apoptosis is a genetically encoded type of programmed cell death that plays a crucial role in both physiological and pathological conditions [37, 38]. Emerging evidence has suggested that the aberrant expression of survival factors protects tumors from cell death following the activation of intrinsic or extrinsic apoptotic pathways especially in oncogenesis [39]. Previous studies have demonstrated that shikonin exerts its antitumor effects by modifying programmed cell death including apoptosis and necroptosis [21, 40]. In this study, we found that shikonin induced early (Annexin V+, PI-) and late apoptosis (Annexin V+, PI+) in HCT116 and SW480 colon cancer cells in a dose-dependent manner. However, no significant proportion of necroptotic cells (Annexin V-, PI+) was identified. In addition, the pan-caspase inhibitor ZVAD was proven to be effective in reducing the proportion of early apoptotic cells, further confirming that shikonin induced apoptosis but no necroptosis in colon cancer cells. The mitochondrial apoptotic pathway has been identified as one of the major apoptotic pathways [15]. Apoptosis proceeds from changes in mitochondrial membrane permeability that are regulated by Bcl-2 family of proteins [41, 42]. In our studies, we found that the mitochondrial membrane potential of HCT116 and SW480 cells was depolarized following treatment with a concentration gradient of shikonin and the depolarization was attenuated by the overexpression of Bcl-2 or Bcl-xL. Meanwhile, shikonin decreased the expression of Bcl-2 and Bcl-xL *in vitro* and *in vivo*. Although the mechanism that underlies the release of mitochondrial apoptotic proteins needs further studies, the Bcl-2 family members play a central role in regulating shikonin-induced apoptosis in colon cancer cells. Apoptosis execution is dependent on the cascading activation of a group of caspases [23]. In this study, we found that shikonin increased activities of caspase 3 and 9 *in vitro* and *in vivo*, which was also attenuated by the overexpression of Bcl-2 or Bcl-xL. Taken together, shikonin induces a Bcl-2 family-based mitochondrial apoptosis program in colon cancer cells.

Compared with normal cells, tumor cells have higher metabolism and oxidative stress and are more vulnerable to ROS induced by exogenous agents [43].Therefore, the elevation of ROS creates a strategy to selectively killing cancer cells [16]. Some chemotherapy drugs exert anti-tumor effects either by direct ROS-generation or by abrogating the antioxidant system [44]. Shikonin has been shown to be a ROS inducer in some cancer cells [18, 31]. We previously reported that shikonin induced the accumulation of ROS in gastric cancer cells, which played an upstream role in the activation of cell apoptosis [11]. The present study showed that shikonin dose-dependently potentiated the generation of intracellular ROS in colon cancer cells *in vitro* and *in vivo*. To further elucidate the mechanisms of ROS in shikonin-induced apoptosis, we assessed two antioxidants, NAC and GSH. Antioxidants potently rescued shikoin-induced cell death and caspase activation. In addition, shikonin-induced depolarization of the mitochondrial membrane potential and decreased expression of Bcl-2 or Bcl-xL were likewise recovered, indicating that ROS acted upstream of the mitochondria in shikonin-induced apoptosis in colon cancer cells. These data suggest that the generation of ROS, down-regulated expression of Bcl-2 and Bcl-xL, depolarization of the mitochondrial membrane potential and activation of the caspase cascade were components of a programmed event shikonin-induced apoptosis in colon cancer cells.

In conclusion, our data showed the ability of shikonin to induce apoptosis of colon cancer cells *in vitro* and *in vivo*. Shikonin-induced apoptosis in colon cancer cells was mediated by the mitochondria and regulated by Bcl-2 family of proteins. The accumulation of intracellular ROS played an upstream role in shikonin-induced apoptosis. In addition, shikonin presented minimal toxicity for non-neoplastic colon cells and no liver injury in xenograft models, showing the efficacy and safety in the growth control of colon cancer cells *in vitro* and *in vivo*. Our results suggested that shikonin can serve as a potential treatment of human colon cancer.

## MATERIALS AND METHODS

### Cell culture and reagents

Human colon cancer cell lines HCT116 and SW480, as well as the normal human colon mucosal epithelial cell line NCM460, were purchased from the Institute of Basic Medical Sciences, Chinese Academy of Medical Sciences. Cells were routinely cultured in RPMI 1640 media (Gibco, NY, USA) containing 10% fetal bovine serum (HyClone, UT, USA) in a cell incubator with an atmosphere of 5% CO_2_ at 37°C.

Shikonin was purchased from Sigma-Aldrich (St. Louis, MO, USA) and was dissolved in DMSO (Sigma-Aldrich, St. Louis, MO, USA). ZVAD-FMK was purchased from MedChemExpress (NJ, USA). N-acetyl-L-cysteine (NAC) and L-glutathione (GSH) were purchased from Beyotime Institute (Jiangsu, China).

### Cell viability and clone formation assays

The MTT assay (Roche, Mannheim, Germany) was determined to detect cell viability according to the manufacturer’s protocol. Briefly, cells were seeded in 96-well plates and were treated with differing concentrations of drugs for a specific time. Next, MTT (5 mg/ml) was incubated for 4 hours and solubilized by DMSO (Sigma, USA) after removing the supernatant. Finally, the absorbance was measured in a microplate reader (Bio-Tec Instrument) at 490 nm. One hundred HCT116 or SW480 cells were plated and treated with an increasing concentration of shikonin for 24 h, and then the culture medium was replaced and cultured for 7 days. The clones were fixed, stained with crystal violet (Sinopharm Chemical Reagent Co. Ltd) and counted.

### Cell cycle analysis

SW480 cells were plated in 6-well plates for 12 h and then were treated with shikonin for 24 h. Cell cycle analysis was performed using a FACS flow cytometer (BD Biosciences, CA) in the presence of Propidium Iodide buffer with RNase (Beyotime Institute, Jiangsu, China).

### Cell apoptosis analysis

HCT116 and SW480 cells were plated in 6-well plates for 12 h and then were treated with the agents for 24 h. Cells were harvested and washed twice with cold PBS. Annexin V FITC and Propidium Iodide (PI) kits (Beyotime Institute, Jiangsu, China) were used to determine the cell death profile using a FACS flow cytometer (BD Biosciences, CA).

### Evaluation of caspase 3/9 activity

Caspase 3/9 activity assay kits (Beyotime Institute, Jiangsu, China) were used to detect caspase activities according to the manufacturer’s protocol. Briefly, cells were plated in 6-well plates for 12 h, treated with agents for 24 h, harvested and resuspended in lysis buffer. Cell protein was normalized, and caspase activities were evaluated by interaction between substrates and enzymes.

### Western blot

Cells or tumor tissues were suspended in Laemmli Buffer (Bio-Rad Laboratory, USA). Protein concentrations were measured and normalized using the BCA Protein Assay Kit (Pierce, USA). Equal amounts of protein samples were electrophoresed by SDS-PAGE and were transferred to polyvinylidene difluoride transfer membranes (Bio-Rad Laboratory, USA). The membrane was blocked with 5% fresh nonfat milk in TBST and was incubated with specific primary antibodies in TBST overnight at 4°C. The membrane was washed 3 times with TBST and incubated with secondary antibodies for 2 hours at room temperature. Finally, the blot bands were visualized using an ECL kit (Bio-Rad, Hercules, CA). The Bcl-2, Bcl-xL, BAX, Caspase 3 and 9 antibodies were from Cell Signaling Technology (MA, USA), and the GAPDH, c-Myc, Cyclin D and Cyclin E antibodies were from Abcam (Cambridge, UK); the goat IgG-HRP secondary antibodies against rabbit and mouse were from Zhongshan Golden Bridge Biotechnology (Beijing, China).

### Measurement of mitochondrial membrane potential

The mitochondrial membrane potential was monitored using a Mitochondrial Membrane Potential Assay Kit (Beyotime Institute, Jiangsu, China) according to the manufacturer’s protocol. Cells were plated in 6-well plates for 12 h, treated with agents for 12 h, harvested and resuspended with JC-1 buffer for 30 min at 37°C in the dark. The mitochondrial membrane potential was quantified using a FACSCalibur flow cytometer (BD Bioscience, CA).

### Measurement of intracellular ROS

Intracellular ROS was measured using an Oxygen Species Assay Kit (Beyotime Institute, Jiangsu, China) according to the manufacturer’s protocol. Cells were plated in 6-well plates for 12 h, treated with agents for 12 h and incubated with DCFH-DA probes at 37°C in the dark. The DCF fluorescence intensity was measured by flow cytometry (BD Bioscience, CA).

### Plasmid transfection

Bcl-2 and Bcl-xL expression plasmids were synthesized by Genechem Institute (Shanghai, China). The overexpression sequences of Bcl-2 and Bcl-xL were constructed in the backbone plasmid GV316. Plasmid transfections were performed using Lipofectamine™ 2000 from Thermo Fisher Scientific (MA, USA) according to the manufacturer’s protocol. Western blot analysis was used to detect the overexpression effect in SW480 cells transiently transfected with overexpressing vectors.

### Establishment of xenografts

All surgical procedures and care were conducted with conformity to the NIH guidelines. Animal experiments were approved by the Animal Care and Use Committee of Chinese PLA Hospital. BALB/c nude male mice (18-22 g in weight, five-to-six weeks old) were purchased from Vital River Laboratory (Beijing, China). Xenografts were established by the subcutaneous injection of 5×10^6^ SW480 tumor cells into the right flanks of mice. When the xenografts were palpable, the tumor-bearing models were administered SHK (0, 3, 6 mg/kg body weight) via gastric infusion once every three days. The tumors were measured every five days. The tumor volumes were calculated using the following formula: volume = 0.5 × length × width^2^. One month later, the mice were sacrificed, and the tumors were removed and weighed. The MDA Assay Kit (Wanlei Biology, Shenyang, China) was utilized to detect the malondialdehyde (MDA) content of xenografts according to the manufacturer’s protocols. The histological toxicity of shikonin in the livers of nude mice was analyzed by hematoxylin and eosin staining.

### Statistical analysis

The values are presented as the means ± SD for at least three independent experiments. GraphPad Prism 6 (GraphPad, San Diego, CA) was used to perform statistical analyses and figure processing. Student’s *t* test and two-way ANOVA were employed for significant difference analysis. A *P* value < 0.05 was considered to be statistically significant.
